# Estimation of Seasonal Risk Caused by the Intake of Lead, Mercury and Cadmium through Freshwater Fish Consumption from Urban Water Reservoirs in Arid Areas of Northern Mexico

**DOI:** 10.3390/ijerph120201803

**Published:** 2015-02-04

**Authors:** Myrna Nevárez, Luz O. Leal, Myriam Moreno

**Affiliations:** 1 Environment and Energy Department, Advanced Materials Research Center (CIMAV) S.C., Miguel de Cervantes 120, Chihuahua, Chih. 31109, Mexico; E-Mail: myrna.nevarez@cimav.edu.mx; 2 Tecnológico de Monterrey, Campus Chihuahua. Av. Heróico Colegio Militar 4700, Chihuahua, Chih. 31302, Mexico; E-Mail: myriamvmoreno@gmail.com

**Keywords:** mercury, cadmium, lead, season, freshwater fish, muscle, provisional tolerable weekly intake (PTWI)

## Abstract

Bioavailability and hence bioaccumulation of heavy metals in fish species depends on seasonal conditions causing different risks levels to human health during the lifetime. Mercury, cadmium and lead contents in fish from Chihuahua (Mexico) water reservoirs have been investigated to assess contamination levels and safety for consumers. Muscle samples of fish were collected across the seasons. Lead and cadmium were analyzed by inductively coupled plasma-optical emission spectrometry, and mercury by cold-vapor atomic absorption spectrometry. The highest concentrations of cadmium (0.235 mg/kg), mercury (0.744 mg/kg) and lead (4.298 mg/kg) exceeded the maximum levels set by European regulations and Codex Alimentarius. Lead concentrations found in fish from three water reservoirs also surpassed the limit of 1 mg/kg established by Mexican regulations. The provisional tolerable weekly intake (PTWI) suggested by the World Health Organization for methyl mercury (1.6 µg/kg bw per week) was exceeded in the spring season (1.94 µg/kg bw per week). This might put consumers at risk of mercury poisoning.

## 1. Introduction

Heavy metals from natural and anthropogenic sources are present in water reservoirs, therefore, high concentrations of them can be found in fish due to their accumulation through the diet and via the gills [1]. Some characteristics of the aquatic environment such as water flow rate, temperature, pH, sunlight and precipitation, among others, which change through the seasons, affect the bioavailability of these pollutants in water and sediments [2,3,4,5,6,7], so a correlation between heavy metals accumulation in fish tissues, fishes dietary habits and heavy metals fraction bioavailable in water and sediments has been reported [8].

Geochemical maps of Chihuahua County (Mexico) show that lead and cadmium are widely distributed in its streams sediments [9]. Anthropogenic sources can also be present due to wet and dry deposition of heavy metals [10] and recreational activities of local residents, such as fishing, boat rides and jet ski use, mainly in Chihuahua and El Rejón water reservoirs. The Chuviscar dam, located near a human settlement, is exposed to garbage and wastewater discharge, while the San Marcos dam is in the proximity of a rural location, where agricultural activities take place.

Excluding accidental and occupational exposure dietary intake is considered the most important source of human exposure to heavy metals causing several health problems [11]. Mercury, lead and cadmium are contaminants associated to fish consumption [12]. Lead poisoning could damage cardiovascular, renal, gastrointestinal, hematological and reproductive systems, as well as cause sub-cellular changes and neuro-developmental disorders, being the latter the most significant [13].

Chronic cadmium exposure causes renal problems due to its accumulation in the proximal tubular cells, decreasing the glomerular filtration rates and eventually leading to renal failure. It also causes skeletal system problems as a secondary effect of renal dysfunction or directly by demineralization owing to its accumulation in bone [13,14]. The cadmium absorbed through intake is bio-accumulated in the liver and kidney and the human body takes from 10 to 30 years to excrete this element [14].

Prenatal exposure through consumption of fish with high concentrations of methyl mercury causes serious diseases, such as cerebral palsy, mental retardation, neurological abnormalities and more infant mortality [1,15].

Given the toxicity and potential health risk, the content of heavy metals in fish muscle has been regulated in Mexico by an Official Mexican Standard [16], establishing the maximum levels for mercury, cadmium and lead in 1, 0.5 and 1 mg/kg wet weight, respectively. The European Food Safety Authority (EFSA) in the Commission Regulation (EC) set the maximum levels for mercury, cadmium and lead in fish muscle in 0.50, 0.050 and 0.30 mg/kg wet weight, respectively [17]. The Codex Alimentarius Commission (WHO, FAO) sets maximum levels for mercury, cadmium and lead in 0.50, 0.1 and 0.2 mg/kg fresh weight, respectively [18,19].

In addition to the established maximum concentration levels of heavy metals, it is extremely important to know their weekly intake, due to its association with health risk. The Provisional Tolerable Weekly Intake (PTWI) is an estimate of the amount of a substance in air, food, soil or drinking water that can be assimilated weekly per unit body weight (bw) over a lifetime without appreciable health risk [20]. The WHO has established a PTWI of 5 µg/kg bw per week for total mercury [21], 5.6 µg/kg bw per week for cadmium [22] and 25 µg/kg bw per week for lead [11].

A previous study was conducted by the authors to evaluate arsenic concentrations in fish from Chihuahua County dams and it was found that the arsenic concentration varied through the sampling periods [23]. However, it should be highlighted that this is the first study done to determine the concentrations of heavy metals (Hg, Cd, Pb) in fish and estimate their weekly intake through the consumption of fish from water reservoirs of Chihuahua County.

There are several techniques for heavy metals determination. Inductively coupled plasma optical emission spectrometry (ICP-OES) is widely employed due to its high sensitivity and multielemental character. The most used technique for mercury determination is cold-vapor atomic absorption spectrometry (CV-AAS), since it provides high sensitivity, selectivity and low operation costs [24].

Therefore, the aims of this study were: (i) to determine the concentrations of mercury, cadmium and lead in fish muscle through different seasons, and (ii) to estimate the weekly intake of these trace metals, comparing them with the PTWI recommended by the WHO [11,21,22].

## 2. Experimental Section

### 2.1. Study Area

**Figure 1:**
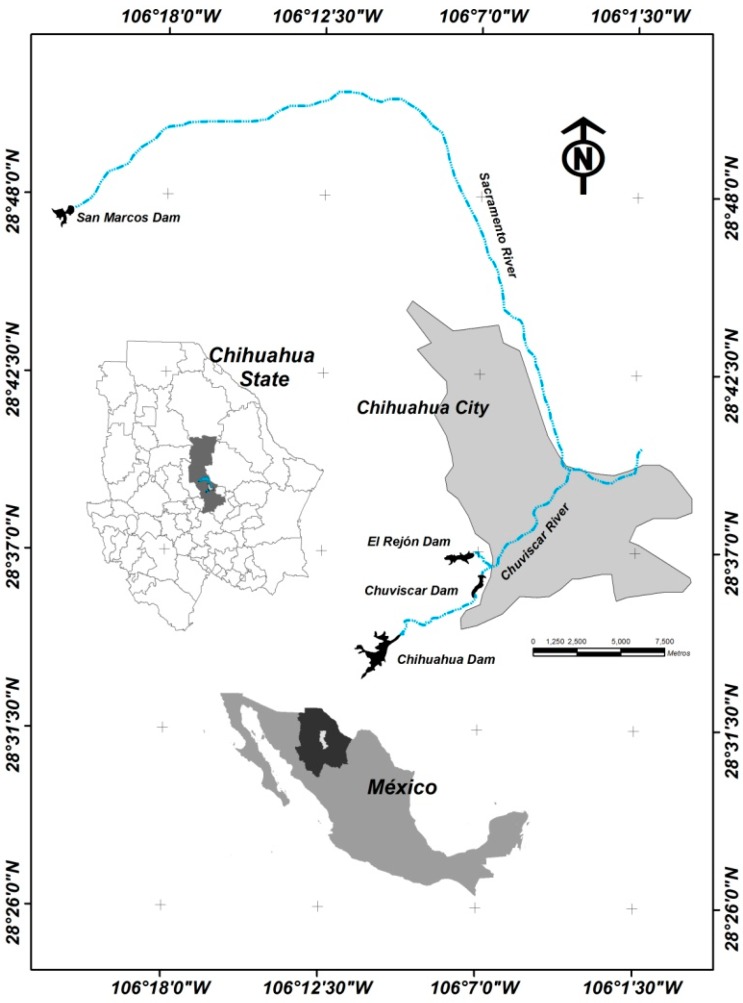
Location map of the water reservoirs in Chihuahua County, Mexico.

The study area is located in Chihuahua County, Mexico (Figure 1). The water reservoirs called Chuviscar, Chihuahua and El Rejón are located within the limits of Chihuahua City. The Chuviscar River is a tributary of these dams, whose storage capacities are 2.1 MCM (millions of cubic meters), 24.83 MCM, and 6.53 MCM respectively. The San Marcos water reservoir, with a storage capacity of 4.45 MCM, is outside the Chihuahua city limits and its tributary is the Sacramento River. The study area belongs to the hydrological region 24, Bravo-Conchos (RH-24), and the water reservoirs are in the basin of the Rio Conchos-El Granero water reservoir. Dams were built originally to supply water to the city of Chihuahua, and currently are being used as flood control and as recreational areas for residents [25]. Fishing in the dams under study is a common activity.

### 2.2. Sampling

Fish were purchased from fishermen who use traditional fishing methods. All sampling was done during the spring, summer, autumn and winter seasons, except for the summer sampling in the Chuviscar water reservoir, where for safety reasons sampling was not carried out. Forty eight specimens were analyzed during this study, 12 in each season, *i.e.*, six of *Ictalurus* *punctatus* and six of the genus Lepomis (four of *Lepomis cyanellus* and two of *Lepomis macrochirus*). The length of each specimen (±10%) was selected according to the criterium suggested by the Guide for Assessment of Chemical Contamination [26]. The analyzed species are the most consumed: green sunfish (*Lepomis cyanellus*) and channel catfish (*Ictalurus punctatus*) from the Chihuahua and El Rejón dams, channel catfish (*Ictalurus punctatus*) from Chuviscar dam and, bluegill (*Lepomis macrochirus*) from San Marcos water reservoir. The fish samples were taken in duplicate. The fish muscle samples were bagged, labeled and kept at 0 °C for later laboratory analysis.

### 2.3. Reagents

Nitric acid, sulfuric acid and hydrogen peroxide, all of analytical grade (J.T. Baker, Center Valley, PA, U.S.A.), were used for acid digestion. Deionized water (18.2 MΩ·cm) was employed for all dilutions. The cadmium and lead standard solutions used for calibration were produced by diluting a certified reference material of 100 mg/L of QCS-27 (High Purity Standards, Charleston, SC, U.S.A.). A solution of 1000 mg/L of Hg (High Purity Standards) was diluted for calibration purposes.

### 2.4. Apparatus

The mercury analysis was carried out by cold vapor-atomic absorption spectrometry (CV-AAS) using an atomic absorption spectrometer (GBC, Avanta Σ, Melbourne, Australia). The measurement was conducted at 253.7 nm, with argon gas flow of 120 mL/min and a pressure of 40 psi flow. Lead and cadmium determinations were performed by inductively coupled plasma-optical emission spectrometry (ICP-OES) (Thermo Jarrell Ash, AP duo, Dortmund, Germany). The measurements were made at 220.353 (117) nm and 228.802 (113) nm for Pb and Cd, respectively. The argon gas flow was of 1 L/min.

### 2.5. Sample Pre-Treatment

Muscle tissue was removed from each fish. These samples were subjected to an open digestion procedure. Acid digestion was performed with HNO_3_-H_2_O_2_-H_2_SO_4_. Nitric acid (HNO_3_, 5 mL) was added to the samples, which were then placed on a hot plate at 70 °C until the disappearance of the characteristic orange fumes of this acid. Later, sulfuric acid (H_2_SO_4_, 5 mL) was added, increasing the temperature to 180 °C for 1 h. Then, the samples were heated up to 315 °C until the disappearance of the characteristic white fumes of this acid and hydrogen peroxide (H_2_O_2_, 1 mL) was finally added. After 30 min, the samples were cooled to room temperature and filtered. Finally, they were diluted up to 50 mL with deionized water. The method was validated using the certified reference material DOLT-3 (Dogfish liver) from the National Research Council of Canada. The same sample pre-treatment described above was also employed for the DOLT-3 sample.

### 2.6. Statistical Analysis

The statistical analysis was carried out for each water reservoir, where season factor was considered. Prior to the statistical analysis, data normality was confirmed using the Ryan-Joiner test. Significant differences between seasons found in the analysis of variance (ANOVA) were evaluated with a Tukey’s test, using the Minitab^®^ 15 statistical software (Minitab Inc., State College, PA, USA).

### 2.7. Estimated Weekly Intake

The estimation of the weekly intake of heavy metals by fish fillet consumption was calculated assuming an average weekly consumption of 245 g [27] multiplied by the average concentration of the target analyte (mercury, cadmium and lead) in fish muscle, and then divided by the average body weight of Chihuahua State residents (75 kg) [28]. The results were interpreted in terms of the WHO provisional tolerable weekly intake for mercury, cadmium and lead.

## 3. Results and Discussion

### 3.1. Method Validation

The analysis of the certified reference material allowed the validation of the method, comparing the results obtained against the certified values of the DOLT-3. Satisfactory recoveries for mercury (99.4%), lead (96.8%) and cadmium (99.6%) were reached, as shown in Table 1.

**Table 1:** Recoveries of certified reference material DOLT-3.

Metal	Certified Value (mg/kg)	Obtained Value * (mg/kg)	Recovery (%)
Mercury **^a^**	3.37 ± 0.14	3.35 ± 0.09	99.4
Lead **^b^**	0.32 ± 0.05	0.31 ± 0.03	96.8
Cadmium **^b^**	19.4 ± 0.6	19.34 ± 0.55	99.6

Notes: * The mean and standard deviation of the obtained value was calculated with ten samples (n=10). **^a^** Cold vapor-atomic absorption spectrometry (CV-AAS); **^b^** Inductively coupled plasma-optical emission spectrometry (ICP-OES).

### 3.2. Metals Concentrations

As can be seen in Table 2, heavy metals concentration in fish fillet showed significant differences (*p* ≤ 0.05) through the sampling season. In addition, some values exceeded both Mexican and European standards.

**Table 2:** Concentration of cadmium, lead and mercury in muscle of fish collected from water reservoirs of Chihuahua County in different seasons **^1^**.

Water Reservoirs	Species	Sampling Season	Cadmium (mg/kg Wet Weight)	Lead (mg/kg Wet Weight)	Mercury (mg/kg Wet Weight)
El Rejón	*Channel catfish*	Spring	0.061 ± 0.013**^a^**	2.327 ± 0.080**^b^**	0.041 ± 0.022
		Summer	0.235 ± 0.013**^b^**	<0.042**^a^**	0.039 ± 0.022
		Autumn	<0.0025**^a^**	3.154 ± 0.080**^c^**	0.059 ± 0.022
		Winter	<0.0025**^a^**	<0.042**^a^**	0.047 ± 0.022
	*Green sunfish*	Spring	0.027 ± 0.014	1.718 ± 0.560**^a,b^**	<0.005**^a^**
		Summer	<0.0025	<0.042**^a^**	0.098 ± 0.012**^b^**
		Autumn	<0.0025	3.516 ± 0.560**^b^**	0.059 ± 0.012**^a,b^**
		Winter	<0.0025	<0.042**^a^**	0.034 ± 0.012**^a,b^**
Chihuahua	*Channel catfish*	Spring	<0.0025	0.729 ± 0.103**^b^**	<0.005**^a^**
		Summer	0.010 ± 0.007	<0.042**^a^**	0.047 ± 0.010**^a,b^**
		Autumn	0.024 ± 0.007	2.110 ± 0.103**^c^**	0.042 ± 0.010**^a,b^**
		Winter	<0.0025	<0.042**^a^**	0.089 ± 0.010**^b^**
	*Green sunfish*	Spring	<0.0025	2.746 ± 0.087**^b^**	<0.005**^a^**
		Summer	0.121 ± 0.055	<0.042**^a^**	0.465 ± 0.256**^b^**
		Autumn	<0.0025	1.525 ± 0.087**^b^**	<0.005**^a^**
		Winter	<0.0025	<0.042**^a^**	<0.005**^a^**
San Marcos	*Blue gill*	Spring	<0.0025**^a^**	0.623 ± 0.329	0.744 ± 0.119
		Summer	0.036 ± 0.0004**^b^**	<0.042	0.333 ± 0.119
		Autumn	<0.0025**^a^**	0.976 ± 0.329	0.395 ± 0.119
		Winter	<0.0025**^a^**	<0.042	0.275 ± 0.119
Chuviscar	*Channel catfish*	Spring	0.090 ± 0.005**^b^**	4.298 ± 0.626**^b^**	<0.005
		Autumn	<0.0025**^a^**	<0.042**^a^**	0.013 ± 0.004
		Winter	<0.0025**^a^**	<0.042**^a^**	<0.005

Notes: **^1^** Expressed as least squares means ± standard error of mean; n = 46; < Below the detection limit of analytical technique; **^a, b, c^** = Different letters in column show statistical differences at a 0.05 confidence level according to the ANOVA and Tukey tests.

#### 3.2.1. Mercury

The green sunfish species showed significant differences through sampling periods (*p* ≤ 0.05) with the highest concentration of mercury in summer (Table 2). This could be attributed to seasonal lipid content trends. According to the U.S. E.P.A. 2000 Guidance for Assessing Chemical Contaminants [26], many fresh water species increase their lipid contents in late summer and early autumn, since lipids are known as good reservoirs for organic contaminants, correlating the lipid content and methyl mercury [29].

An explanation of this trend could be that methyl mercury has lipophilic properties [30] and it is the most abundant mercury chemical species in fish, accounting for 80% of the total mercury [31]. The average mercury concentrations in all seasons (Table 2) did not exceed the maximum permissible concentration of 1 mg/kg for total mercury established by Mexican regulations [16]. However, the European Commission Regulation in the Official Journal of the European Union [17] and the Codex Alimentarius [18] have set a maximum mercury concentration of 0.50 mg/kg wet weight. Considering this, the bluegill from San Marcos water reservoir exceeded such a limit in the spring season, since a concentration of 0.744 ± 0.199 mg/kg was found. These high mercury concentrations found in San Marcos dam could relate to the contribution of volcanic soil and rock erosion [30] from Majalca Mountains. The Sacramento River, which feeds the San Marcos water reservoir, begins in the Majalca Mountains, which according to Ferriz are a volcanic cauldron [32].

#### 3.2.2. Cadmium

Cadmium behavior showed high concentrations in spring and summer, when a high temperature period (mean of 35 °C), just before the rainy season, could contribute to those results. Thereafter, the cadmium concentration decreased to levels that are not detected by the analytical technique (Table 2). Cadmium bioaccumulation has been associated with high temperatures mainly due to factors such as disturbances in muscle homeostasis [33] and an increase of cadmium concentration in the metallothionein-like proteins [34].

The rainy and dry seasons affected the accumulation, distribution and mobilization of cadmium, since higher concentrations were observed in the dry season, while in the rainy season the addition of organic and inorganic particles and a higher oxygenation can be associated with low cadmium bioavailability [6].

Several authors have reported a decrease of cadmium concentration in water after the rainy season. It was associated to several factors such as dilution by rainfall and runoff [4,5]. The water storage capacity in the water reservoirs of Chihuahua County is increased considerably in rainy season, *i.e.*, El Rejón water reservoir, from 34% of storage capacity in June, increased to 90% in October, and Chihuahua dam, from 46% of storage capacity in June, increased to 92% in October [35].

The reduction of cadmium content in fish muscle after the rainy season was also reported in a study carried out under controlled conditions, where fish were exposed to concentrations of cadmium in water and then transferred to clean water, showing a decrease in accumulated cadmium in muscle [36]. This can be explained since the cadmium accumulation mainly occurs in liver and kidney [36,37,38].

Cadmium concentrations (Table 2) did not exceed the maximum permissible limit of 0.5 mg/kg established by Mexican regulations [16]. However, considering the maximum levels for cadmium concentration (0.1 mg/kg and 0.050 mg/kg wet weight) set by Codex Alimentarius Commission (1989) [18] and the European Commission Regulation in the Official Journal of the European Union (2006) [17] respectively, some of the concentrations found in spring and summer exceeded such limits.

#### 3.2.3. Lead

Lead showed a similar seasonal behavior in most of the water reservoirs (Table 2), presenting the highest concentrations in the autumn season (*p* ≤ 0.05), just after the rainy season. Several authors agree that the contribution of lead to aquatic environments is mainly caused by deposition in rain [39,40,41] or through run-off from soils [42]. The atmospheric dispersion of lead from mine waste has also been reported [43]. In fact, Chihuahua State is the second more important place in Mexico for lead extraction, with an annual production of 56,253 tons in 2008 [44]. Later, a decreasing behavior of lead in other sampling periods is observed. This could be explained by the fact that lead absorption in fish reaches equilibrium only after several weeks of exposure, and it is accumulated in gills, liver, kidney, and bones [45,46,47,48].

The average lead concentration in fish from most of the water reservoirs in spring and autumn season (Table 2) surpassed the permissible limit of 1 mg/kg established by Mexican regulations [16]. Considering the maximum level of lead concentration, 0.30 mg/kg wet weight and 0.2 mg/kg wet weight, set by the European Commission Regulation in the Official Journal of the European Union (2006) [17] and Codex Alimentarius Commission (2001) [19], respectively, all spring concentrations and almost all autumn concentrations exceeded such limits.

### 3.3. Estimation of Weekly Intakes of Hg, Cd and Pb through Fish Consumption

The estimation of heavy metal weekly intakes through consumption of fish is presented in Table 3. As can be seen, the calculated values did not exceed the Provisional Tolerable Weekly Intake (PTWI) suggested by the WHO as a total amount of a substance in air, food, soil or drinking water. The WHO has established a PTWI of 5 µg/kg bw per week for total mercury [21], 5.6 µg/kg bw per week for cadmium [22] and 25 µg/kg bw per week for lead [11]. However, considering the highest values reported for lead and mercury, their weekly intake contributed with a 56% and 49% to the PTWI, respectively, which would be a high contribution through solely consumption of fish, despite of the low average consumption of the local population in comparison with other countries [49].

Subsistence populations have an average consumption of 142.4 g per day [26], four times higher than the average consumption of 245 g per week for the local population. Estimation of weekly intake of subsistence populations for lead and mercury (57 and 9.9 µg/kg/bw/week, respectively) exceeded the PTWI recommended by the WHO. The PTWI of 5 µg/kg bw per week for total mercury [21], was not exceeded regardless of the season (Table 3). As has been reported, the estimated percentage of methyl mercury in fish fillet corresponds to 80% of total mercury [31]. Thus, the PTWI for methyl mercury in bluegill from San Marcos water reservoir in the spring season is 1.94 µg/kg bw per week, which exceed the PTWI of 1.6 µg/kg bw per week for methyl mercury suggested by the WHO [31].

The cadmium provisional tolerable weekly intake of 5.6 µg/kg bw per week allowed by the WHO [22] was not exceeded, regardless of the season (Table 3).

The lead provisional tolerable weekly intake of 25 µg/kg bw per week [11] was not surpassed regardless of the season (Table 3). However, the estimated PTWI for lead exposure through intake has been associated with a decrease of at least three intelligence quotient (IQ) points in children and an increasing of systolic blood pressure of approximately 3 mmHg (0.4 kPa) in adults.

**Table 3:** Estimated weekly intake for cadmium, lead and mercury (µg/kg/bw/week) through fish consumption of local population.

Water Reservoir	Species	Sampling Season	Cadmium	Lead	Mercury
El Rejón	*Channel catfish*	Spring	0.199	7.602	0.134
		Summer	0.768	NA	0.127
		Autumn	NA	10.303	0.193
		Winter	NA	NA	0.154
	*Green sunfish*	Spring	0.088	5.612	NA
		Summer	NA	NA	0.320
		Autumn	NA	11.486	0.193
		Winter	NA	NA	0.111
Chihuahua	*Channel catfish*	Spring	NA	2.381	NA
		Summer	0.033	NA	0.154
		Autumn	0.078	6.893	0.137
		Winter	NA	NA	0.291
	*Green sunfish*	Spring	NA	8.970	NA
		Summer	0.395	NA	1.519
		Autumn	NA	4.982	NA
		Winter	NA	NA	NA
San Marcos	*Blue gill*	Spring	NA	2.035	2.430
		Summer	0.118	NA	1.088
		Autumn	NA	3.188	1.290
		Winter	NA	NA	0.898
Chuviscar	*Channel catfish*	Spring	0.294	14.040	NA
		Autumn	NA	NA	0.042
		Winter	NA	NA	NA

Note: NA: Not available due concentration in fillet fish was below the analytical technique limit.

Several studies have also characterized the risk for heavy metals intake through fish consumption. Vieira *et al.* [50] reported a PTWI for total Hg of 2.866 and 1.911 µg/kg bw for groups of children from 1–3 and 4–6 years old, respectively, due to the consumption of horse mackerel collected from the Atlantic Ocean in Portuguese waters. Although the PTWI found did not exceed the WHO recommended value, they mentioned that considering the 85% of methyl mercury in the total Hg content, the PTWI for that particular contaminant would be exceeded. However, the Cd and Pb intake did not surpass the suggested limits of the WHO.

A total mercury weekly intake of 1.791 µg/kg·bw was reported as a result of marine and freshwater fish and canned fish product consumption in Serbia. This intake was estimated taking into account a consumption of 340 g/week [51].

The PTWI for total mercury found in the consumption of albacore (6.55 µg/kg·bw) and rosefish (5.25 µg/kg·bw), both species from the Adriatic Sea, surpassed the WHO recommended value. Nevertheless, the PTWI values for cadmium (0.04–0.25 µg/kg·bw) and lead (0.04–4.96 µg/kg·bw) did not exceed it [52].

It has been also reported that the consumption of 150 g of either louvar or swordfish, acquired from fish retailers in Madrid (Spain), exceeded the recommended value of PTWI for mercury in women in childbearing age, whereas for Cd and Pb it did not [53]. This is an important issue, considering the human health threats associated with methyl mercury exposure through intake [1,15] affecting public health, mainly in children [54].

## 4. Conclusions

Significant differences were found in the concentrations of mercury, lead and cadmium in fish fillets through the different seasons. The highest lead concentration found in fish muscle exceeded by up to four times the permissible limit of 1 mg/kg established by Mexican regulations, 14 times the maximum level of 0.30 mg/kg wet weight set by the Commission Regulation EC No 1881/2006 of the European Community and more than 21 times the maximum level of 0.2 mg/kg established by Codex Alimentarius. The highest cadmium concentration exceeds by almost five times the maximum level of 0.050 mg/kg wet weight set by Commission Regulation EC No 1881/2006 of the European Community and by more than two times the maximum level of 0.1 mg/kg set by Codex Alimentarius.

The Provisional Tolerable Weekly Intake (PTWI) of heavy metals suggested by The World Health Organization (WHO) was not surpassed, but in spring season lead and mercury contributed significantly. The PTWI for the estimated methyl mercury was exceeded in the spring season in San Marcos dam. Thus, the exposed population, especially pregnant women, are at serious risk associated to health effects. An estimate of fish consumption among the local population as well as sports fishermen is suggested.
